# Defining the Role of Nuclear Factor (NF)-κB p105 Subunit in Human Macrophage by Transcriptomic Analysis of *NFKB1* Knockout THP1 Cells

**DOI:** 10.3389/fimmu.2021.669906

**Published:** 2021-10-13

**Authors:** Domenico Somma, Fatma O. Kok, David Kerrigan, Christine A. Wells, Ruaidhrí J. Carmody

**Affiliations:** ^1^ Centre for Immunobiology, Institute of Infection, Immunity & Inflammation, College of Medicine, Veterinary and Life Sciences, University of Glasgow, Glasgow, United Kingdom; ^2^ Centre for Stem Cell Systems, The University of Melbourne, Melbourne, VIC, Australia

**Keywords:** NF-κB, macrophage, NFKB1, transcriptomics, toll-like receptors, THP1 cells

## Abstract

Since its discovery over 30 years ago the NF-ĸB family of transcription factors has gained the status of master regulator of the immune response. Much of what we understand of the role of NF-ĸB in immune development, homeostasis and inflammation comes from studies of mice null for specific NF-ĸB subunit encoding genes. The role of inflammation in diseases that affect a majority of individuals with health problems globally further establishes NF-ĸB as an important pathogenic factor. More recently, genomic sequencing has revealed loss of function mutations in the *NFKB1* gene as the most common monogenic cause of common variable immunodeficiencies in Europeans. *NFKB1* encodes the p105 subunit of NF-ĸB which is processed to generate the NF-ĸB p50 subunit. *NFKB1* is the most highly expressed transcription factor in macrophages, key cellular drivers of inflammation and immunity. Although a key role for *NFKB1* in the control of the immune system is apparent from *Nfkb1^-/-^* mouse studies, we know relatively little of the role of *NFKB1* in regulating human macrophage responses. In this study we use the THP1 monocyte cell line and CRISPR/Cas9 gene editing to generate a model of *NFKB1^-/-^* human macrophages. Transcriptomic analysis reveals that activated *NFKB1^-/-^* macrophages are more pro-inflammatory than wild type controls and express elevated levels of *TNF*, *IL6*, and *IL1B*, but also have reduced expression of co-stimulatory factors important for the activation of T cells and adaptive immune responses such as *CD70*, *CD83* and *CD209*. *NFKB1* ^-/-^ THP1 macrophages recapitulate key observations in individuals with *NFKB1* haploinsufficiency including decreased *IL10* expression. These data supporting their utility as an *in vitro* model for understanding the role of *NFKB1* in human monocytes and macrophages and indicate that of loss of function *NFKB1* mutations in these cells is an important component in the associated pathology.

## Introduction

Common variable immunodeficiency (CVID) is the most common primary immuno-deficiency in humans and is a clinically and genetically heterozygous disorder characterised by increased susceptibility to infection, hypogammaglobulinaemia and poor responses to vaccination. Recent advances in genome sequencing have led to the identification of genetic determinants of CVID in a significant number of clinical cases. These analyses have identified loss of function variants in the gene encoding the NF-ĸB p105 subunit (*NFKB1*) as the most common monogenic cause of CVID in Europeans (1–3). In these studies loss of function variants of *NFKB1* had the highest probability of disease association and explained the largest number of CVID cases. CVID resulting from *NFKB1* loss of function is characterised by lymphadenopathy, splenomegaly, and autoimmunity, as well as an increased incidence of cancers including solid tumours and hematologic malignancies. In addition to loss of function mutations, monogenic heterogeneous mutations affecting the nuclear localisation and stability of NF-ĸB1 can also lead to immune-pathologies including autoinflammatory diseases such as Behçet disease (4).

The NF-ĸB family of transcription factors are critical regulators of immune homeostasis and the inflammatory response. NF-ĸB is activated by virtually all immunoreceptors, including cytokine-, antigen- and Toll-like- receptors, and co-ordinates the expression of more than 500 genes encoding pro-inflammatory factors such as cytokines and chemokines, as well as genes controlling cell survival, proliferation, and differentiation. There are five NF-ĸB subunits, RelA, c-Rel, p50, RelB and p52, which form homo-and heterodimers to regulate gene transcription. The NF-ĸB p50 subunit is generated from the limited proteasomal processing of the p105 precursor protein which also functions as an inhibitor of NF-ĸB activation. p50 lacks a transactivation domain and so may act as a transcriptional repressor as well as activator depending on whether it forms homodimers or heterodimers with other NF-ĸB subunits (5, 6).

Studies of individuals with loss of function *NFKB1* variants reveal significant effects on immune cell development and function. These analyses show that *NFKB1* haploinsufficiency leads to reduced invariant natural killer cell numbers as well as reduced CD4 and CD8 T cells at early stages of development, while leading to an accumulation of effector memory T cells. B cell homeostasis is similarly disrupted leading to an overall B cell deficiency including reduced memory and class switched B cells (3). B cells with *NFKB1* loss of function mutations also show impaired proliferation following B cell receptor activation or stimulation with Toll-like receptor (TLR) ligand and display reduced IgG production *ex vivo* (3). In contrast, stimulation of *NFKB1* deficient whole blood with TLR ligand results in elevated expression of the pro-inflammatory cytokines IL-1β and TNFα (7). A recently identified homozygous mutation in *NFKB1* (G960R) led to perturbations in both T- and B-cell maturation and function, reduced CD4+ memory T cell numbers, while T cell responses to mitogens and cytokine secretion were reduced. These studies also revealed skewed T-cell receptor repertoire, and reduced output of naïve T cells in the *NFKB1* variant patient (8).

*NFKB1* haplo-insufficiency phenotypes have some overlap with those of the *Nfkb1^-/-^* mouse. Similar to *NFKB1* loss of function variants in humans, *Nfkb1* ^-/-^ mice are more susceptible to bacterial infection and have significantly reduced peripheral B cell numbers. In mice, *Nfkb1* is important for mature B cell survival but does not appear to have a significant role in B cell development. However, when *Nfkb1* is deleted in combination with *Rel* or *Nfkb2* B cell development is abnormal (9–12). *Nfkb1* ^-/-^ B cells also proliferate poorly in response to TLR stimulation but, in contrast to *NFKB1* haplo-insufficient B cells, proliferate normally in response to B cell receptor activation (13). In contrast to the apparent role for *NFKB1* in T cell maturation in humans, in mouse *Nfkb1* appears dispensable for normal T cell maturation but is required for the development of Th2 responses involving IL-4, IL-5 and IL-13 (14, 15). These differences suggested that further study of NF-ĸB1 function in human cells is warranted to more completely understand its role in the regulation of immune responses.

*NFKB1* is the most abundantly expressed transcription factor in macrophages (16), key initiators of chronic inflammatory disease. In addition to regulating TLR-induced pro-inflammatory gene expression in activated macrophages, *NFKB1* also plays a key role in shaping macrophage polarisation and innate immune memory responses (17). Innate immune memory refers to the adaptation of innate immune cells to re-activation with the same or different stimulus and can result in enhanced or reduced inflammatory responses (18). *Nfkb1* ^-/-^ macrophages are defective in LPS tolerance, a form of innate immune memory characterized by reduced expression of pro-inflammatory cytokines such as *Tnf (* 19). Loss of this anti-inflammatory function in *Nfkb1* ^-/-^ mice results in chronic low-grade inflammation leading to accelerated aging and a shortened life span (20). Conversely, pro-inflammatory *Nfkb1^-/-^* macrophages adopt an anti-tumour phenotype that reduces the tumour burden in mouse models of colitis associated cancer (21). Despite the recognized physiological roles of *NFKB1* in macrophages, the consequences of *NFKB1* deficiency in human macrophages is not known. Here we describe an *in vitro* model cell system for the study of *NFKB1* function in human monocytes and macrophages using CRISPR/Cas9 gene editing to generate a *NFKB1^-/-^* THP1 human monocyte cell line. Deletion of *NFKB1* does not alter the activation of the NF-ĸB pathway in response to TLR stimulation, however transcriptomic analysis reveals a critical role for *NFKB1* in regulating key pro-inflammatory genes including co-stimulatory factors of T cell mediated adaptive immune responses.

## Results

### *NFKB1^-/-^* THP1 Macrophages

**Figure 1:**
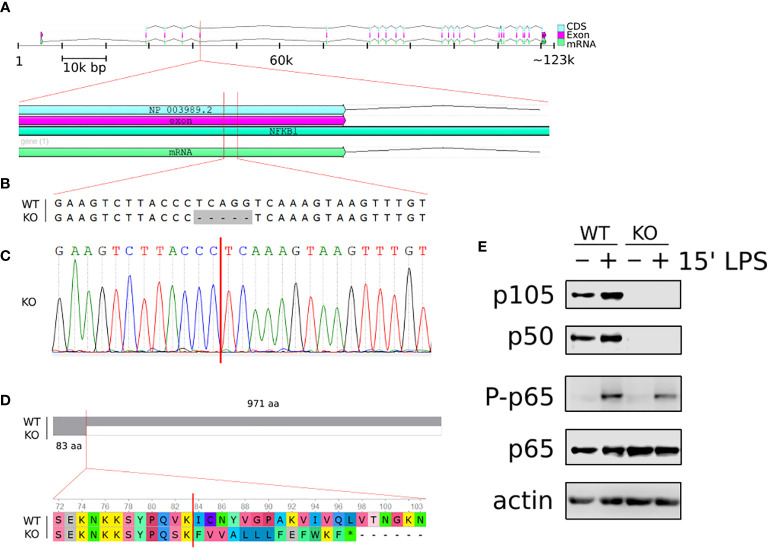
Generation of *NFKB1* ^-/-^ THP1 cells. **(A)** Schematic overview of homo sapiens *NFKB1* gene organisation on chromosome 4; cDNA sequence (CDS), exon and mRNA are indicated. **(B)** Alignment of *NFKB1* exon 5 DNA sequences of wild type (WT) and *NFKB1^-/-^* (KO) cells showing CRISPR/Cas9 generated deletion. **(C)** Sanger sequencing chromatogram of exon 5 in *NFKB1^-/-^* cells containing a frameshift mutation. **(D)** Alignment of *NFKB1* open reading frames in WT and *NFKB1* ^-/-^ cells, grey area indicates sequence identify. **(E)** PMA-induced macrophages from WT and *NFKB1^-/-^* THP1 cells were stimulated with LPS (10 ng/mL) for 15 min, and p105/p50 (*NFKB1* protein products) analysed by western blot. Antibodies specific for p65 phosphorylation at S536 (p-p65) were used to measure NF-κB pathway activation, anti- p65 and anti-actin antibodies were used as loading controls.

To explore the role of p105/p50 in the regulation of human macrophage responses we employed CRISPR/Cas9 gene editing approaches to generate a *NFKB1* ^-/-^ THP1 human monocyte cell line. THP1 is a human monocytic cell line that can be differentiated into macrophages by phorbol myristate acetate (PMA) and is an established *in vitro* model for the study of human monocyte and macrophage responses (22). Targeted CRISPR/Cas9 guided double strand breaks generated a 5 base pair deletion in exon 5 of the *NFKB1* gene which created a premature stop codon (**Figures 1A** **–D**). The absence of p105/p50 protein was confirmed by western blot analysis (**Figure 1E**).

PMA-induced differentiation of NFKB1^-/-^ THP1 cells to macrophage was indistinguishable to that observed for wild type (WT) THP1 cells (data not shown) confirming that NFKB1 is not required for macrophage differentiation as previously reported in Nfkb1^-/-^ mice (19). To investigate the functional response of NFKB1^-/-^ THP1 derived macrophages to inflammatory stimuli we next stimulated cells with the Toll-like receptor (TLR) 4 ligand lipopolysaccharide (LPS). First, we assessed the impact of NFKB1 knockout on the activation of the NF-ĸB pathway as measured by phosphorylation of the p65 subunit of NF-ĸB at S536 by the IKK complex, an event strongly linked to NF-ĸB activation (23). This revealed similar levels of p65 S536 phosphorylation in LPS stimulated NFKB1^-/-^ cells when compared to WT controls (**Figure 1E**), in line with data from Nfkb1^-/-^ mice demonstrating that NFKB1 is not required for IKK activation in the NF-ĸB pathway. Collectively, these data show that deletion of *NFKB1* in THP1 does not affect PMA induced differentiation to macrophages, or LPS-induced activation of the NF-ĸB pathway, thereby demonstrating their suitability for investigating role of *NFKB1* in LPS-stimulated cells.

### *NFKB1* Deletion Significantly Alters the Transcriptional Profile of THP1 Macrophages

**Figure 2:**
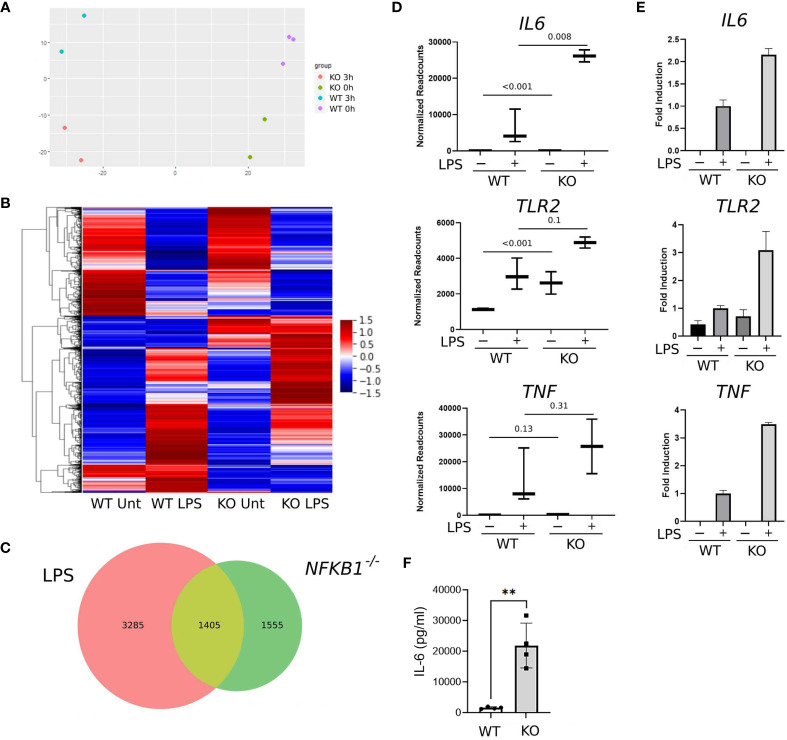
*NFKB1* deletion alters the transcriptional profile of THP1 macrophages. **(A)** Principal component analysis plot showing sample similarity of RNAseq analysis of control (WT) and *NFKB1* ^-/-^ (KO) cells untreated (Unt) or treated with LPS. **(B)** Heatmap showing differentially expressed genes, Z-score normalized. Relative expression is shown as the average of replicates ranging from maximum to minimum values. **(C)** Venn diagram showing the overlap of LPS-inducible genes and genes differentially expressed in *NFKB1* ^-/-^ cells relative to WT cells in unstimulated and LPS stimulated conditions (p-value and q-value < 0.05). **(D)** Selected gene expression data plotted as normalized RNAseq readcounts. Data presented as mean ± SD of biological replicates, p values adjusted for Benjamini-Hochberg are indicated. **(E)** Patterns of expression for selected genes confirmed in an independent experiment using QPCR. Data presented as mean ± SEM of technical replicates. **(F)** PMA-induced macrophages from WT and NFKB1-/- THP1 cells were stimulated with LPS, and the supernatant was analysed by IL-6 ELISA. (n = 4, data presented as mean ± SD of four independent experiments). **p < 0.003 using T-test analysis.

To determine the role of *NFKB1* in regulating inflammatory gene expression we stimulated PMA induced *NFKB1* ^-/-^ and WT THP1 macrophages with LPS for 3 hours prior to analysis by RNA-seq alongside untreated controls. Unsupervised principal component analysis revealed distinct transcriptional profiles for WT and *NFKB1* ^-/-^ cells in both unstimulated and LPS-stimulated conditions (**Figure 2A**). This analysis showed that the main variance between experimental groups was LPS stimulation and *NFKB1* deletion (**Figure 2A**).

Overall, our analysis identified 4,690 genes differentially expressed in LPS treated THP1 cells when compared to untreated controls (**Figure 2C**). Comparison of WT and *NFKB1^-/-^* cells identified 2,960 genes differentially expressed in *NFKB1* ^-/-^ cells relative to WT controls. Of this *NFKB1* signature gene set, 1,405 genes were also found to be LPS regulated, indicating that 30% of genes induced by LPS are controlled by *NFKB1* and 47% of *NFKB1*-regulated genes are induced by LPS (**Figures 2B, C**). Of note, the direction of regulation of genes by *NFKB1* was consistent across experimental conditions and there is little overlap between genes up-regulated and genes down-regulated in *NFKB1^-/-^* cells either in steady state or LPS-stimulated conditions (**Supplemental Figure S2**). We validated the RNA-seq data by performing an independent QPCR analysis of selected genes including *IL6*, *TLR2*, and *TNF* which are known to be regulated by *NFKB1* (**Figures 2D** **, E**). The increased expression of *IL6* in *NFKB1* ^-/-^ cells relative to WT controls was further validated by ELISA analysis of cell supernatants (**Figure 2F**). Together these data demonstrate that *NFKB1* may both positively and negatively regulate the expression of hundreds of genes, both in the steady state and upon LPS stimulation, consistent with previously described dimer-specific roles of the NF-ĸB p50 subunit (5).

### *NFKB1^-/-^* THP1 Macrophages Have an Inflammatory Phenotype

**Figure 3:**
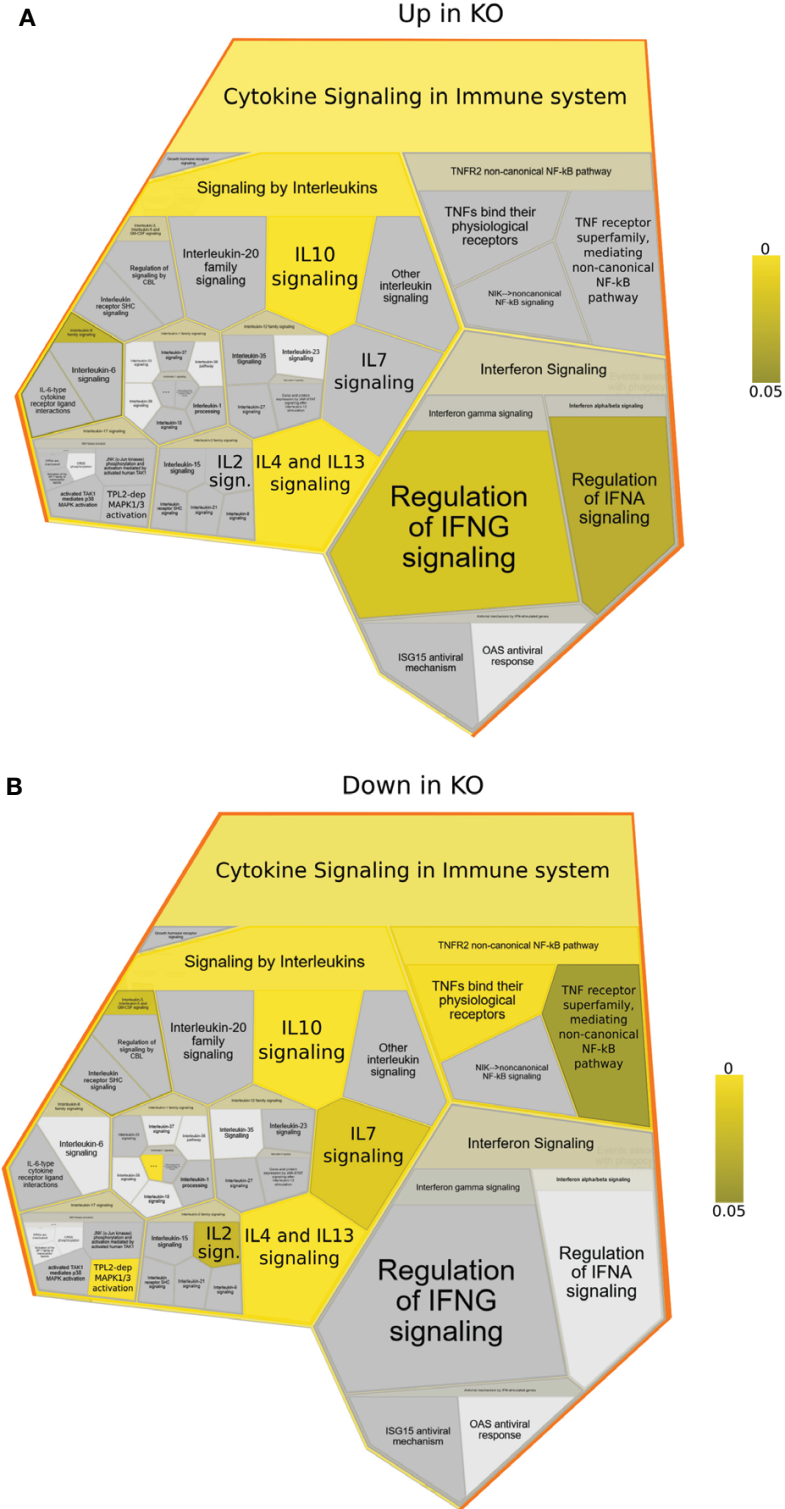
Reactome foam plots identifying enriched pathways using genes **(A)** upregulated or **(B)** downregulated in *NFKB1* ^-/-^ (KO) macrophages (either untreated or LPS treated) compared to wild type controls. Yellow bar indicates p values.

**Figure 4:**
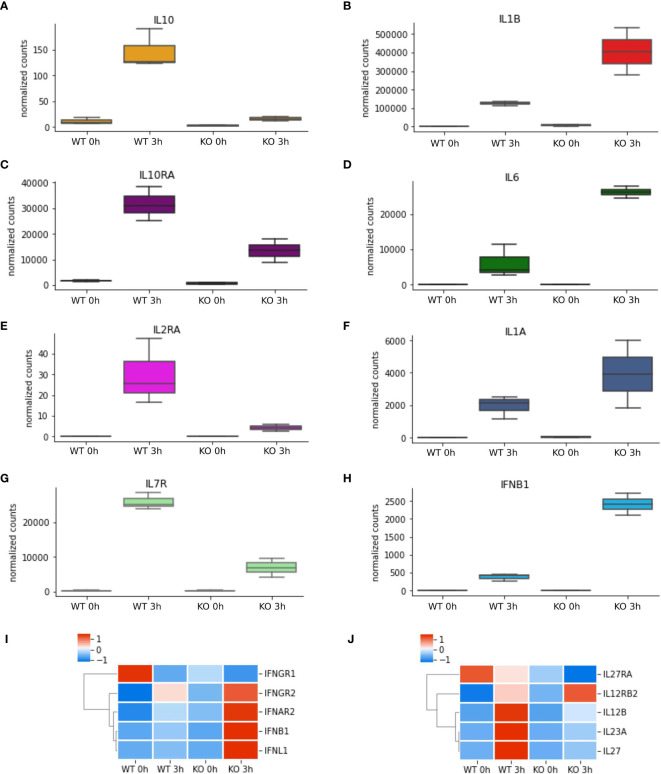
*NFKB1* ^-/-^ macrophages have an inflammatory phenotype. **(A–H)** RNAseq data of selected differentially expressed cytokine and cytokine receptor genes identified by Reactome pathway analysis. Heatmaps of differentially expressed **(I)** interferon cytokines and receptors and **(J)** IL-12-family of cytokines and receptors from RNAseq analysis, Z-score normalized.

To better understand which signalling pathways are regulated by *NFKB1* we used the manually curated Reactome database to identify over-represented signalling pathways. We divided the genes in two subsets; one composed of *NFKB1*-dependent LPS-induced genes and the other of genes downregulated in *NFKB1^-/-^* cells (**Supplemental Figure S2B**). Reactome analysis of these two gene sets revealed that *NFKB1* controls a macro “Signalling by Interleukins” pathway incorporating a number of key immuno-receptor initiated pathways (**Figure 3**, **Supplemental Tables S1** and **S2**; **Supplemental Figure S3**). These pathways include the IL-10 signalling pathway (**Supplemental Figure S4**), due to the reduced expression of *IL10* and *IL10RA* in LPS-stimulated *NFKB1^-/-^* cells compared to WT controls (**Figure 4A**). In contrast, expression of the inflammatory cytokine genes *IL6*, *IL1B* and *IL1A* is increased in *NFKB1^-/-^* cells relative to WT controls (**Figure 4**). In addition, reduced expression of *IL4R* in LPS-stimulated *NFKB1^-/-^* cells relative to WT controls also implicates *NFKB1* in the regulation of IL-4 and IL-13 activated responses (**Supplemental Figure S5**). Similarly, reduced expression of *IL2RA* and *IL7R* in LPS-stimulated *NFKB1^-/-^* cells relative to WT controls implicates *NFKB1* in the regulation of IL-2 and IL-7 activated responses (**Figure 4**). Increased expression of components of the interferon response, including *IFNB1*, *IFNL1*, and *IFNAR2*, in LPS-stimulated *NFKB1^-/-^* cells relative to WT controls also identifies *NFKB1* as a regulator of interferon pathways (**Figure 4**).

Another macro pathway identified by this analysis as significantly modified in *NFKB1^-/-^* cells is “Chemokine receptors bind chemokines” (**Supplemental Figure S6** and **Supplemental Tables S1**, **S2**). Here, LPS-induced increased the expression of the chemokines *CXCL1*, *CCL1*, *CXCL3*, *CXCL8*, *CXCL16* and *CCL20*, and reduced the expression of the chemokine receptors *CCR2*, *CXCR2*, *CXCR4*, *CCR7*, and of chemokines *CCL5*, *CCL19* and *CCL22* in *NFKB1^-/-^* cells relative to WT controls and identifies *NFKB1* as a regulator of chemokine signalling.

**Figure 5:**
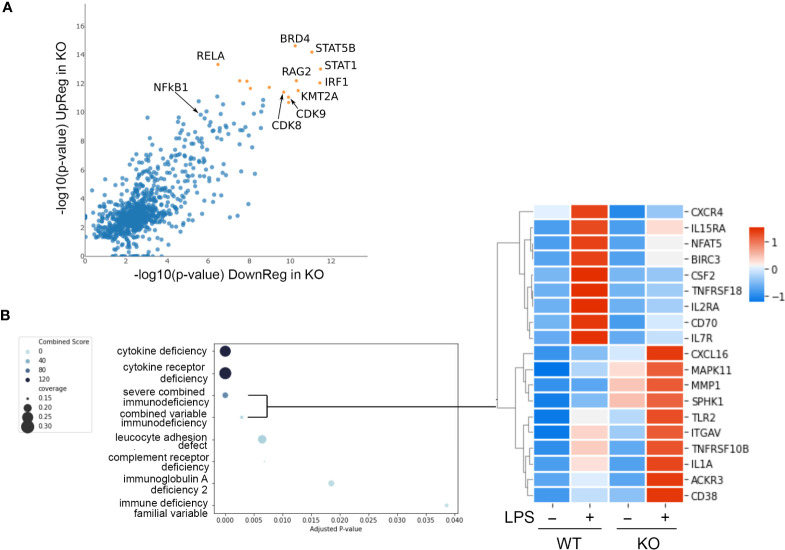
*NFKB1* transcription factor network and disease associations. **(A)** Lisa analysis identifying transcription factors associated with the expression LPS dependent of genes differentially expressed in *NFKB1* ^-/-^ cells (p < 0.01). Top most significant factors indicated by orange symbols. **(B)** Identification of disease associated gene signatures in the GeneRif database with significant similarity to the transcriptional signature of LPS-stimulated *NFKB1* ^-/-^ cells (q value < 0.05). Heatmap on right shows selected genes associated with the severe combined and common variable immunodeficiency disease signatures, Z-score normalized.

Reactome is a manually curated peer-reviewed database covering almost 11,000 human genes but excludes ~38% of the genes differentially expressed in our analysis. To get further insight into our full dataset we next used Enrichr to query the BioPlanet database which integrates several pathway databases (24). This pathway analysis confirmed the findings of the Reactome analysis such as the involvement of *IL-4, IL-2, IL-1* and *IL-10* pathways, but also highlighted some additional pathways such as those activated by the *IL-12* family cytokines (*IL12B, IL23A, IL27, IL27RA* and *IL12RB2*) (**Figure 5**), as well as components of *EGF, VEGF, TGF* cytokine signalling (**Supplemental Figure S7** and **Supplemental Table S3**) as altered in *NFKB1* ^-/-^ cells. Together this data demonstrates that deletion of *NFKB1* alters not only the expression of key inflammatory cytokines such as *IL1B*, *IL6*, *IL10* and *IFNB1*, but also the expression of cytokine and chemokine receptors.

### Identifying a *NFKB1* Transcription Factor Network and Disease Associations

Transcription factors form complex networks to regulate gene expression by interacting with other transcription regulators (25). To explore this aspect of *NFKB1* mediated transcriptional responses we employed Lisa (epigenetic Landscape *In Silico* deletion Analysis) to identify potential cofactors of *NFKB1*. This approach uses DNase-seq and H3K27ac ChIP-seq profiles to identify the transcription factors and chromatin regulators responsible for the control of a differentially expressed gene set. This analysis confirmed the p50 and RelA NF-ĸB subunits as transcription factors associated with genes differentially expressed in LPS-stimulated *NFKB1* ^-/-^ cells relative to WT controls (**Figure 5A**). This analysis also identified the IRF1 and STAT1 transcription factors as co-regulators of these genes, supporting previous reports of a functional interaction between p50 and these factors (26, 27). In addition, this analysis also identified STAT5B, BRD4, RAG2, KMT2A and CDK8/9 as factors associated with *NFKB1* regulated genes, indicating further complexity of the NF-κB1 transcriptional network. Similar findings were obtained in an independent analysis using the TRRUST database (**Figure S8**).

We next evaluated the potential association of the *NFKB1* ^-/-^ transcriptional signature (genes differentially expressed in LPS stimulated *NFKB1* ^-/-^ cells compared to WT controls) with disease associated gene signatures in humans. To perform this we used the GeneRif database of manually curated datasets describing gene signatures associated with human disease. This analysis revealed a significant similarity of the *NFKB1* ^-/-^ transcriptional signature with “Severe combined immunodeficiency” and “Common variable immunodeficiency” disease signatures (**Figure 5B** and **Table S4**). This finding suggests that loss of *NFKB1* function in macrophages, and indeed other innate immune cells such as dendritic cells, is also likely to contribute the common variable immunodeficiency phenotype resulting from *NFKB1* haploinsufficiency.

**Figure 6:**
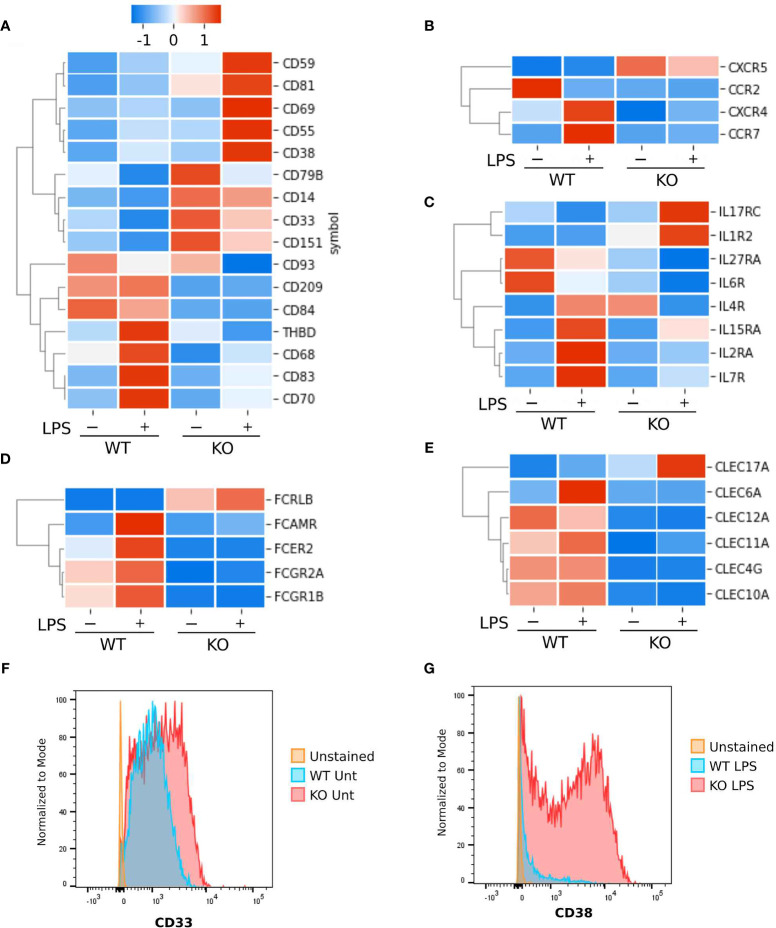
Surface markers differentially expressed between *NFKB1* ^-/-^ and wild type THP1 cells. Heatmaps Z-score normalized showing gene expressions as the average of replicates. **(A)** Cluster differentiation markers. **(B)** Chemokine and cytokines receptors. **(C)** Interleukin receptors. **(D)** Fc receptors. **(E)** C-type lectin domain family members. **(F)** Analysis by flow cytometry of CD33 and **(G)** CD38 cell surface protein expression in untreated (unt) or LPS stimulated wild type (WT) or *NFKB1* ^-/-^ (KO) THP1 cells using specific antibodies.

A key function of innate immune cell activation is co-stimulation of adaptive immune cells. To explore this aspect of *NFKB1* ^-/-^ macrophage responses we examined co-stimulatory gene expression in *NFKB1* ^-/-^ compared to WT controls (**Figure 6**). This revealed that expression of co-stimulatory factors such as CD70, CD83 and CD209 (**Figure 6A**) are decreased in LPS stimulated *NFKB1* ^-/-^ cells when compared to WT controls. In addition, there is also a reduction in Fc receptor gene expression (**Figure 6D**), as well as the CLEC family of genes (**Figure 6E**). These findings, along with the reduced expression of genes encoding interleukin receptors, CCR and CXCR families (**Figure 6B**) mentioned described above, indicate that in addition to a defective acute inflammatory response in *NFKB1* ^-/-^ cells, these cells are also likely to be defective the co-stimulation of T cells. Interestingly, *NFKB1* ^-/-^ cells have altered gene expression in steady state conditions including the anti-inflammatory *CD33* (**Figure 6A**) which results in increased CD33 surface expression in *NFKB1^-/-^* cells as measured by flow cytometry. (**Figure 6F**). Conversely genes encoding inflammatory proteins such as *CD38* (28) are more highly expressed in *NFKB1* ^-/-^ macrophage following LPS stimulation (**Figure 6A**) leading to increased expression of cell surface CD38 protein as confirmed by flow cytometry (**Figure 6G**). The increased expression of CD38 in *NFKB1* ^-/-^ cells has a two-phase distribution, with CD38+ high and low cells evident.

## Discussion

The recent identification of mutations in *NFKB1* leading combined variable immuno-deficiencies further cements the importance of the NF-ĸB transcription factors in the control of inflammation and immunity. However, these findings also highlight that despite a number of studies using animals deficient for *Nfkb1*, the specific impact of loss of *NFKB1* on transcriptional responses in immune cells remains largely undefined. In this study we have generated a model for the study of *NFKB1* deficiency using CRISPR/Cas9 gene editing in the THP1 cell line, an established model for the study of human monocytes and macrophages. Transcriptomic analysis of TLR4 activated THP1 differentiated macrophages revealed a critical role for *NFKB1* in the regulation of TLR4-induced transcriptional responses. This analysis confirms the key role of NF-κB1 in regulating TLR induced transcriptional responses in macrophages while identifying new gene targets of *NFKB1* that may play a role in the pathology associated with *NFKB1* haploinsufficiency.

Overall, our data shows that the expression of pro-inflammatory cytokines such as *TNF*, *IL6, IL1B, IL10* and *IFNB* in *NFKB1* ^-/-^ THP1 macrophages show a similar pattern to that seen in studies of *Nfkb1^-/-^* mice, demonstrating that the biological functions *NFKB1* are conserved across species. Moreover, our analysis of *NFKB1* ^-/-^ THP1 macrophages recapitulates key observations in individuals with *NFKB1* haploinsufficiency including decreased *IL10* expression. These data support the utility of *NFKB1* ^-/-^ THP1 macrophages as a model of *NFKB1* deficiency in human monocytes and macrophages.

A comparison of our data with those reported from studies of *Nfkb1* ^-/-^ mice reveals important differences in patterns of *NFKB1* regulated gene expression between mouse and human cells. This is highlighted by the expression patterns of chemokines which are differentially affected by deletion of *NFKB1* in mouse and human macrophages. While studies of *Nfkb1* ^-/-^ mouse macrophages identified a role for *Nfkb1* in promoting the expression of CCL2 and CCL17 (17), our analysis indicates that *NFKB1* does not significantly regulate their expression in THP1 derived macrophages. Instead, our analysis identifies increased expression of the chemokines CXCL1, CCL1, CXCL3, CXCL8, CXCL16, and CCL20, and decreased expression of CCL5, CCL19 and CCL22 in LPS stimulated *NFKB1* ^-/-^ THP1 cells. While the reasons for these disparities are unclear, they may underline potential differences in chemokine regulation between human and mouse macrophages (29). What is clear however, is that *NFKB1* plays an important role in coordinating the expression of chemokines and their receptors following TLR activation. Indeed, the reduced expression of CCR7 in *NFKB1* ^-/-^ cells reported here would suggest that cell migration to lymph nodes is likely to be impaired in individuals with *NFKB1* haploinsufficiency, which may contribute to the observed pathology. The regulation by *NFKB1* of genes encoding immuno-receptors in addition to chemokine receptors, such as cytokine receptors (IL6R, IL2RA, IL10RA, IL7R, IL4R, IL1R1/2), C-type lectins, Fc receptors, and co-stimulatory molecules emphasises the important role of NF-κB1 in coordinating inflammatory responses. Further understanding of the role of NF-κB1 in these processes will require *in vivo* experimental techniques that can assess cell extrinsic effects not possible with the *in vitro* approaches used in this study.

*NFKB1* may influence target gene expression positively or negatively through p50 heterodimers or homodimers respectively, and also acts as an IκB protein due to the presence of C terminal ankyrin repeat domains (5). In addition, p50 may also regulate transcriptional responses through interaction with other transcription factors such as IRFs and STATs (30–32). Our analysis shows that the *NFKB1* controlled transcriptome significantly overlaps with those controlled by the transcription factors IRF1, STAT5, STAT5B as well the transcriptional regulators BRD4, RAG2, KMT2A, and CDK8/9. This indicates a potential role of *NFKB1* in a number of transcriptional networks and may further explain the significant impact of *NFKB1* deficiency on TLR inducible responses.

Studies of *Nfkb1* knockout mice have identified different roles for NF-κB1 within different immune cell types. Notably, mice heterozygous for *Nfkb1* do not develop the primary immunodeficiency found in humans with *NFKB1* haploinsufficiency (3), suggesting key differences in the role of *NFKB1* in humans and mice. Our data demonstrate the utility of *NFKB1* ^-/-^ THP1 cells as an *in vitro* model for understanding the role of NF-κB1 in human monocytes and macrophages. THP1 cells have been extensively used for the study of monocyte and macrophage functions. Studies to date have demonstrated that the responses of THP1 cells activated with a variety of stimuli are similar to those of primary human monocytes, although the magnitude of cytokine secretion and gene expression changes may differ (22). Understanding the role of NF-κB1 in other immune cells such as T or B cells and the similarities and differences with animal models requires further study in the context of human cells. Although, generating specific mutations found in *NFKB1* variant patients using similar approaches would provide a more accurate model of *NFKB1* variant induced CVID that might provide further insight, this study indicates an important role for monocytes and macrophages in the immunological phenotype of *NFKB1* haploinsufficiency.

## Material and Methods

### Generation of NFKB1^-/-^ THP1 Using CRISPR/Cas9

THP-1 cells were cultured in RPMI containing 10% FBS with of 2 mM glutamine and 100 units/mL penicillin/streptomycin. For generation of CRISPR/Cas9 *NFKB1* knock-out cells, THP1 cells were transfected with a ribonucleoprotein (RNP) complex using the Neon electroporator (ThermoFisherScientific). 2µl (20pmol) of sgRNA (: 5’-ACAAACUUACUUUGACCUGA-3’, Synthego) plus 1µl of Cas9 nuclease (20pmol) (Synthego) was added to 9.5µl Neon buffer T (ThermoFisherScientific) and incubated at room temperature for 10 minutes. THP1 cells were washed with Mg^2+^ and Ca^2+^ free phosphate buffered saline and re-suspended in Neon buffer T at a density of 2 × 10^7^ cells/ml. 7µl of RNP complex was transfected into 10µl of cells using a 10µl Neon Transfection tip (cat# MPK2025) and settings of 1400v, 20msec, and 2xpulse. Serial dilution of cells was performed to isolate single cell clones in 96-well plates. For clone screening, genomic DNA was extracted from cells using the DNeasy Blood and Tissue Kit (QIAGEN) according to the manufacturer’s instruction. DNA was amplified by PCR using primers (F: 5’-ACCTGGCTTTTTAGCCATATCT-3’; R: 5’-TTCAGCTTAGGAGCGAAGGC-3’) and Hot-StarTaq Master Mix Kit (QIAGEN) according to the manufacturer’s instructions. Initial screens were performed by HindIII (NEB) restriction digest of PCR products purified using QIAquick PCR Purification Kit (QIAGEN), according to the manufacturer’s instructions. Gene editing of selected clones was confirmed by Sanger sequencing (GATC Biotech).

### Western Blot

Whole cell lysates were generated from cells suspended in RIPA buffer containing 50 mM Tris–HCl (pH 7.4), 1% Nonidet P-40, 0.25% deoxycholate, 150 mM NaCl, 1 mM EDTA, 1 mM PMSF, 1 mM NaF, 1 mM Na3VO4, 2 µg/ml aprotinin, 1 µg/ml pepstatin and 1 µg/ml leupeptin. After an additional 15 min on ice, cell extracts were centrifuged for 10 min at 14,000 g at 4°C, and supernatants. Protein concentration of lysates were determined using a Bradford assay (Bio-Rad). Denatured samples were subjected to SDS-PAGE, transferred to nitrocellulose membranes, and blocked for 1 h at room temperature using 5% fat free milk powder in PBS–Tween20. Membranes were immunoblotted with specific antibodies. p105/p50 (#12540) and anti-p65 phospho-S536 (#3031), were purchased from Cell Signalling Technologies, p65 (A301-824A) was purchased from Bethyl Laboratories, beta-actin (#A5441) was purchased from Sigma.

### ELISA

IL-6 ELISA (Life Technologies) was performed according to the manufacturer’s instruction using supernatants from THP1 differentiated macrophages either untreated or stimulated for 24 hours with 100 ng/ml LPS.

### Flow Cytometry

THP1 differentiated macrophages were stimulated 24h with LPS 100 ng/ml for 24 hours harvested after trypsin digestion, centrifuged at 400g for 5 min, washed and resuspended with FACS buffer (BD Biosciences), and transferred to FACS tubes (BD Biosciences). Fixable Viability Dye eFluor 780 (eBioscience) was added at 1:1,000 in PBS and incubated for 20 min at 4°C. Cells were then washed with FACS buffer were stained with antibodies specific for either CD33 or CD38 (Biolegend), conjugated with Alexa Fluor 700. Staining was performed in a final volume of 100 μl with antibody dilution 1:100 for 30 min on ice. Cells were washed twice with FACS buffer and resuspended in a final volume of 300 μl, filtered through a 100-μm cell strainer and analysed using a FACS Fortessa (BD Biosciences). Live macrophages were gated based on viability dye. All data generated were analysed using FlowJo software (TreeStar).

### RNA Extraction and QPCR

For RNA-sequencing and real-time quantitative PCR, total RNA was extracted from cells using RNeasy kits (QIAGEN), according to the manufacturer’s instructions and quantified using NanoDrop 1000 Spectrophotometer (ThermoFisher Scientific). 1 µg of isolated RNA was retro-transcribed using random primers and nanoScript reverse transcription kit (Primer Design). QPCR was performed with SYBR Green SuperMix (PerfeCTa #733-1386) using QuantiTect Primer Assays (TNF #QT00029162, IL6 #QT00083720, TLR2 #QT00236131, TBP #QT00000721) using QuantStudio 7 Flex Real-Time PCR System (ThermoFisher Scientific). All data were normalised to TBP. Gene expression changes were calculated using the 2-ΔΔCT method.

### RNAseq

Triplicate independently generated samples for each condition were sequenced to a read depth of 20 million using an Illumina NextSeq™500 platform, generating single-end 75 bp reads fastq files. Original raw data can be found in the NCBI Gene Expression Omnibus database with the accession number GSE162015. For the analysis the University of Glasgow Galaxy (33) server (v18.09) was used and a detailed analysis pipeline is available at http://heighliner.cvr.gla.ac.uk/u/dsomma1/h/hnfkb1-wt-vs-ko. Briefly, we use FastQC (v0.72), MultiQC (v1.5.0) and Trimmomatic (v0.36.5) for FastQ files quality control check, improvement, and plots. FastQ files were mapped on human genome (Ensembl GRCh38.p13 sm primary assembly) using HISAT2 (v2.1.0). Features were assigned using Infer Experiment (v2.6.4) and featureCounts (v1.6.0.3) against human genome annotation (Ensembl GRCh38.98 filtered to contain only protein coding genes, for a total n of genes: 19976). RNAseq reads are good quality (**Figure S1A**), with ~90% of reads mapped uniquely (**Figure S1B**), and 75% assigned to the corresponding gene (**Figure S1C**). Two samples were excluded because PCA identify them as outliers (**Figure S1D**). DESeq2 (v2.11.40.2) was used to determine DE genes. Heatmaps and plots were generate using Python 3.7.4, Jupyter notebook 6.0.4, Pandas 0.23.4, Seaborn 0.10.1. Heatmap of differentially expressed genes in different replicates is shown in **Figure S1E**.

### Gene Enrichment and Pathway Analysis of Differentially Expressed Genes

Genes differentially expressed between WT and *NFKB1^-/-^* cells were filtered for q value < 0.05; to be sure to identify all genes controlled by LPS. LPS-induced genes (comparing WT unstimulated *vs* WT LPS) were filtered for a q value < 0.1, and intersected with the lists previously generated, as indicated in Venn diagram (**Figure S2B**). For pathway analysis Reactome pathway database (34) was used (v71), while gene set enrichment was performed using EnrichR (35) through gseapy (v0.9.16) to query BioPlanet (24), TRRUST (36) and GeneRif (37) databases. To infer transcriptional regulators involved in gene expression Lisa (38) software website was used; top 500 genes (either upregulated or downregulated) ordered by q values were selected and LPS-dependent-p105-independent genes were used as background removal. Specifically, for Reactome, TRRUST and Lisa, genes were split into upregulated and downregulated in *NFKB1^-/-^* to dissect the contribution of each pathway and transcription factor. For BioPlanet and GeneRif analysis upregulated, and downregulated genes were used as one list to provide a broader view.

## Data Availability Statement

The datasets presented in this study can be found in online repositories. The names of the repository/repositories and accession number(s) can be found in the article/**Supplementary Material**.

## Author Contributions

DS, CW, and RC designed research. DS, DK, FK, and RC performed research. DK contributed new reagents. DS and CW analysed data. DS and RC wrote the paper. All authors contributed to the article and approved the submitted version.

## Funding

This work was supported by the Medical Research Council (MR/M010694/1) and Biotechnology and Biological Sciences Research Council (BB/M003671/1 and BB/T007427/1).

## Conflict of Interest

The authors declare that the research was conducted in the absence of any commercial or financial relationships that could be construed as a potential conflict of interest.

## Publisher’s Note

All claims expressed in this article are solely those of the authors and do not necessarily represent those of their affiliated organizations, or those of the publisher, the editors and the reviewers. Any product that may be evaluated in this article, or claim that may be made by its manufacturer, is not guaranteed or endorsed by the publisher.
