# Supplementary material for: Defining the Role of Nuclear Factor (NF)-κB p105 Subunit in Human Macrophage by Transcriptomic Analysis of NFKB1 Knockout THP1 Cells
Source: Front Immunol. 2021 Oct 13;12:669906. doi: 10.3389/fimmu.2021.669906 (PMC8548695; doi:10.3389/fimmu.2021.669906)
Supplement: Supplementary file 1 [file DataSheet_1.pdf]

Supplementary Material

1 Supplementary Figures

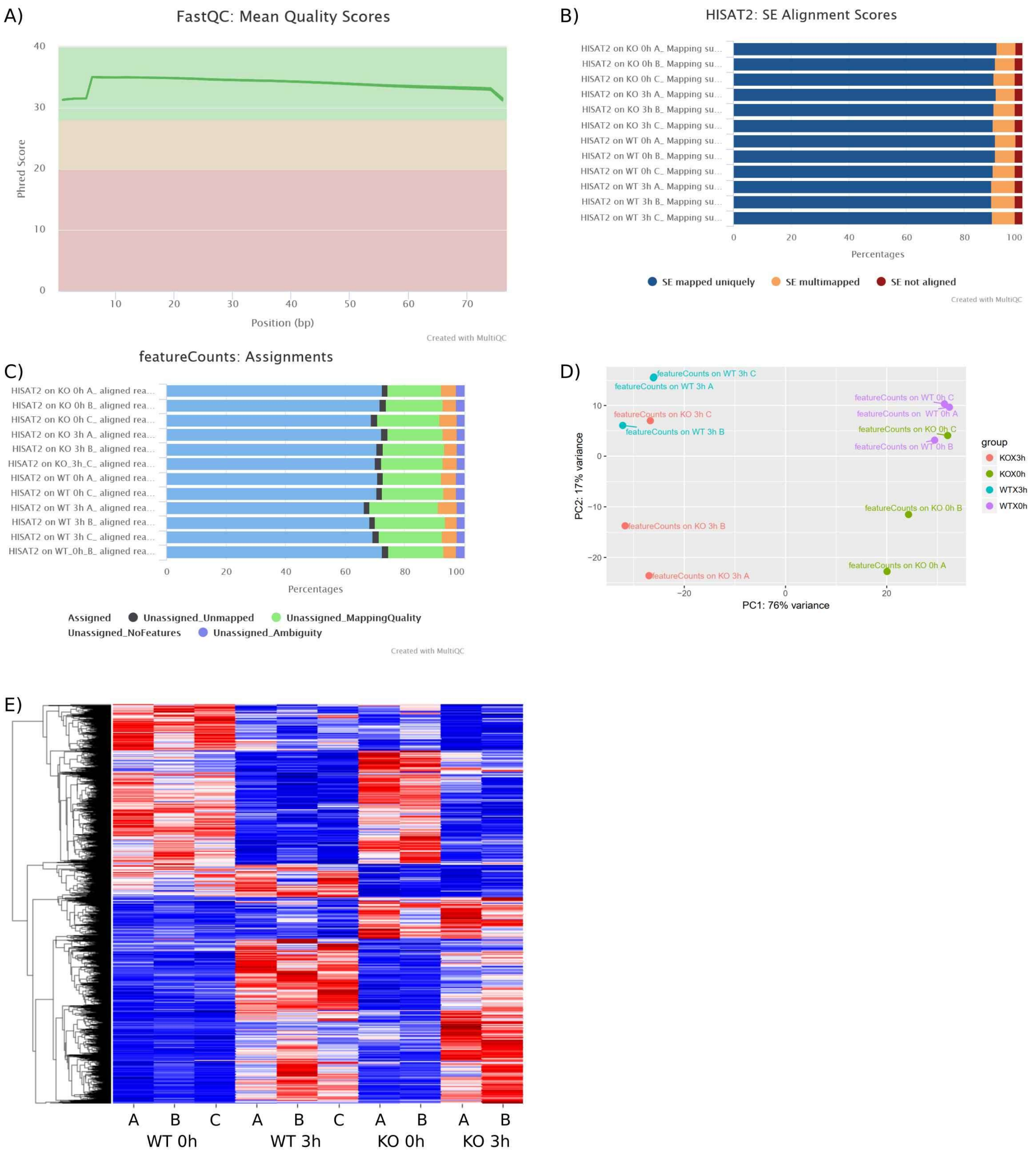

Supplementary Figure 1. RNAseq quality control. (A) RNAseq reads quality. (B) Percentage of reads mapped on human genome and (C) gene assignment. (D) Replicates PCA plot. (E) Heatmap of DE genes in different replicates, Z-score normalized.

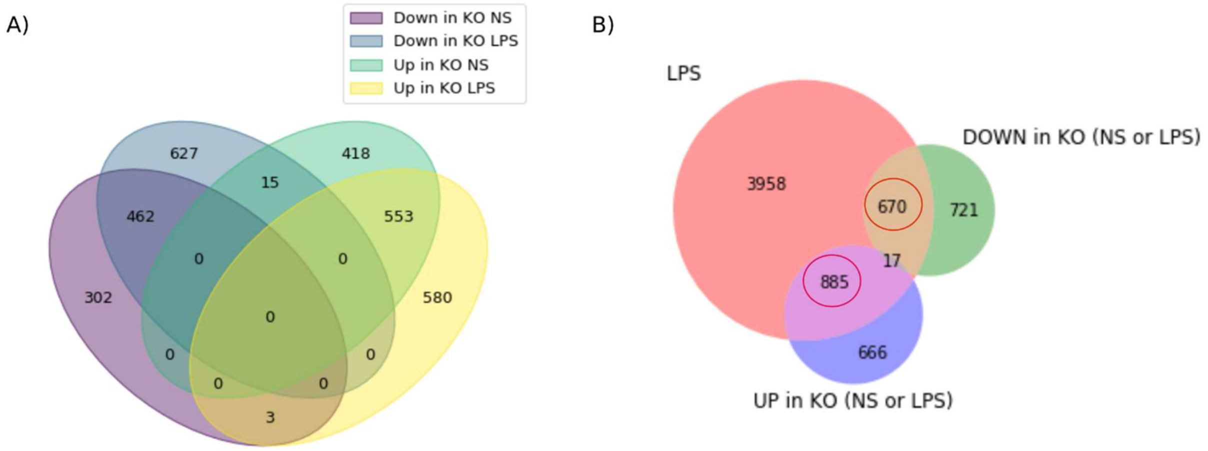

Supplementary Figure 2. (A) Venn diagram showing genes differential expressed overlap among downregulated/upregulated in NFKB1-/- THP1 cells not stimulated or stimulated with LPS (qvalues < 0.05). (B) Venn diagram showing overlap among genes induced by LPS stimulation and genes downregulated/upregulated in NFKB1-/- THP1 (either not stimulated or LPS stimulated); genes is red circles are used for Reactome/EnrichR analysis. LPS-induced genes q values < 0.1, genes upregulated/downregulated in KO q values < 0.05.

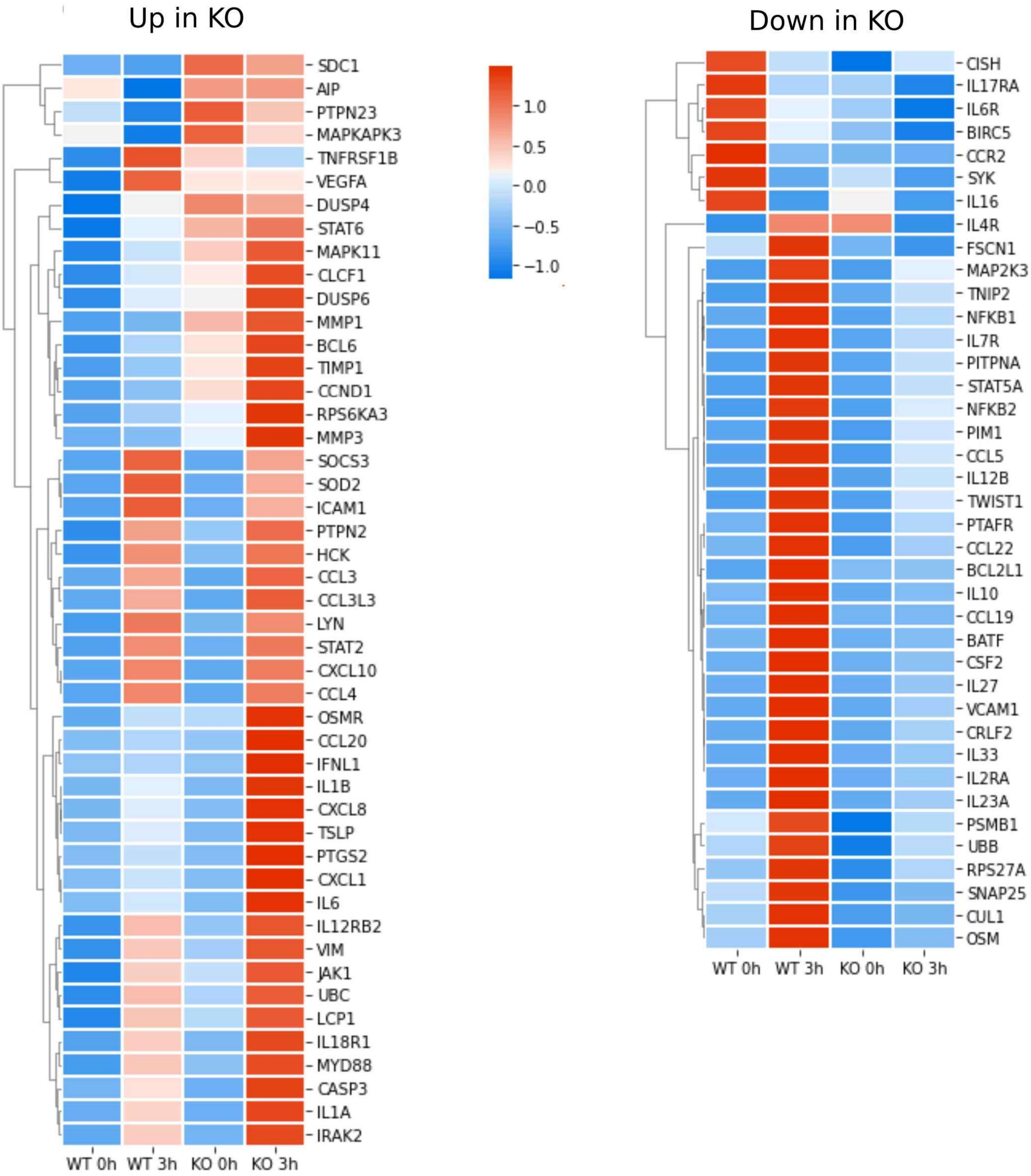

Supplementary Figure 3. Heatmaps showing genes significantly (qvalue < 0.05) upregulated or downregulated in NFKB1-/- THP1 (either not stimulated or LPS stimulated) according to Reactome "Signaling by Interleukins" pathway, Z-score normalized.

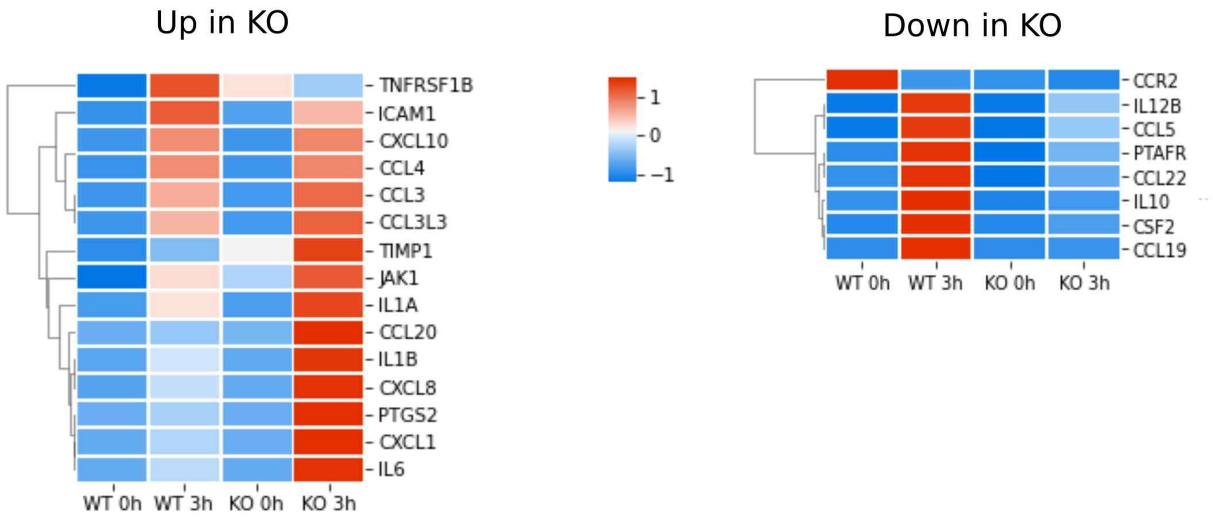

Supplementary Figure 4. Heatmaps showing genes DE (qvalue < 0.05) involved in interleukin-10 Reactome pathway, either upregulated or downregulated in NFKB1$^{-/-}$ THP1, Z-score normalized.

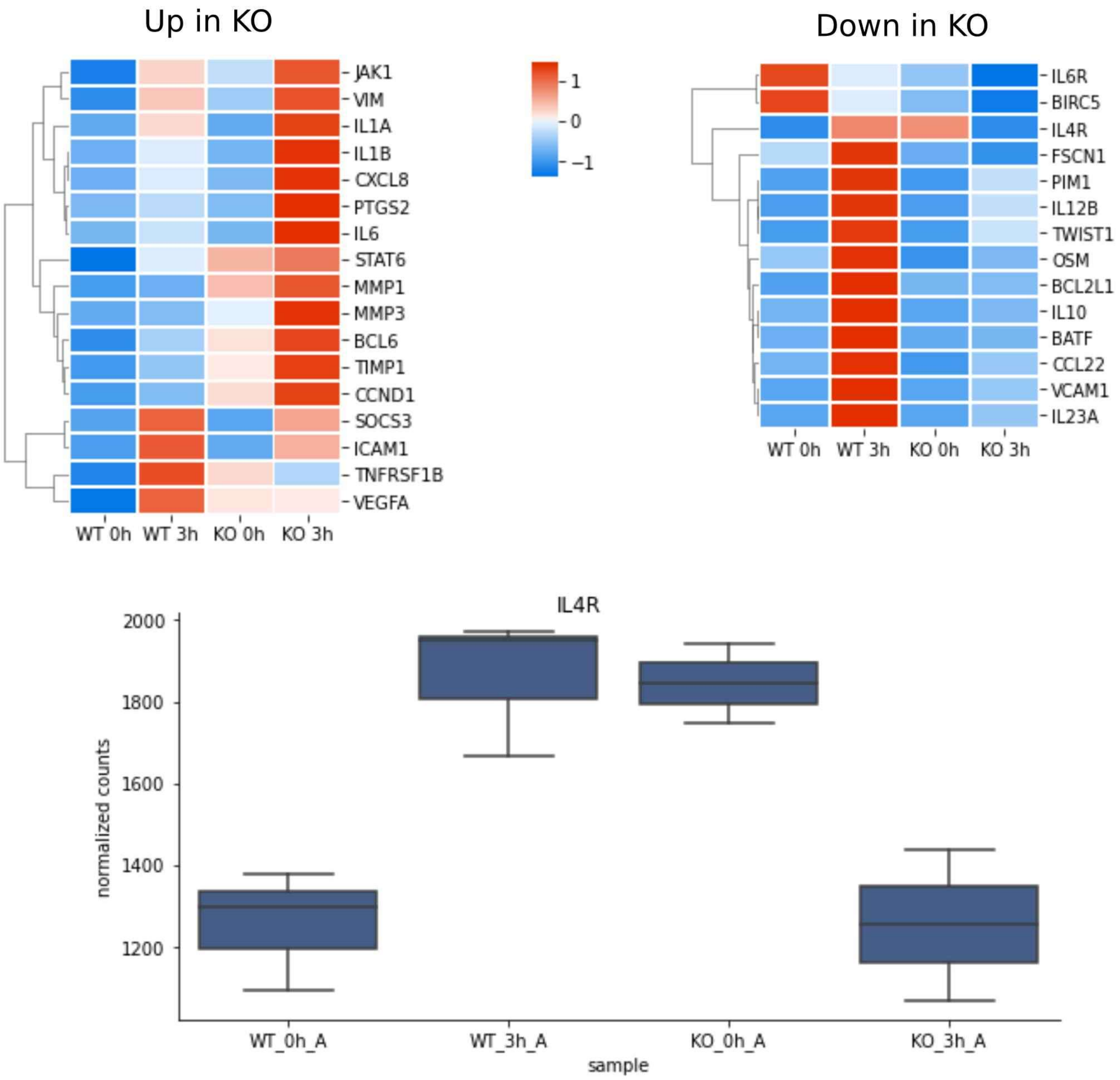

Supplementary Figure 5: Upper panel) Heatmaps showing genes participating in "Interleukin-4 and Interleukin-13" Reactome pathway, either significantly (qvalue < 0.05) upregulated or downregulated in NFKB1$^{-/-}$ THP1, Z-score normalized. **Lower panel)** RNAseq data showing IL4R changes in NFKB1 WT/KO unstimulated or stimulated.

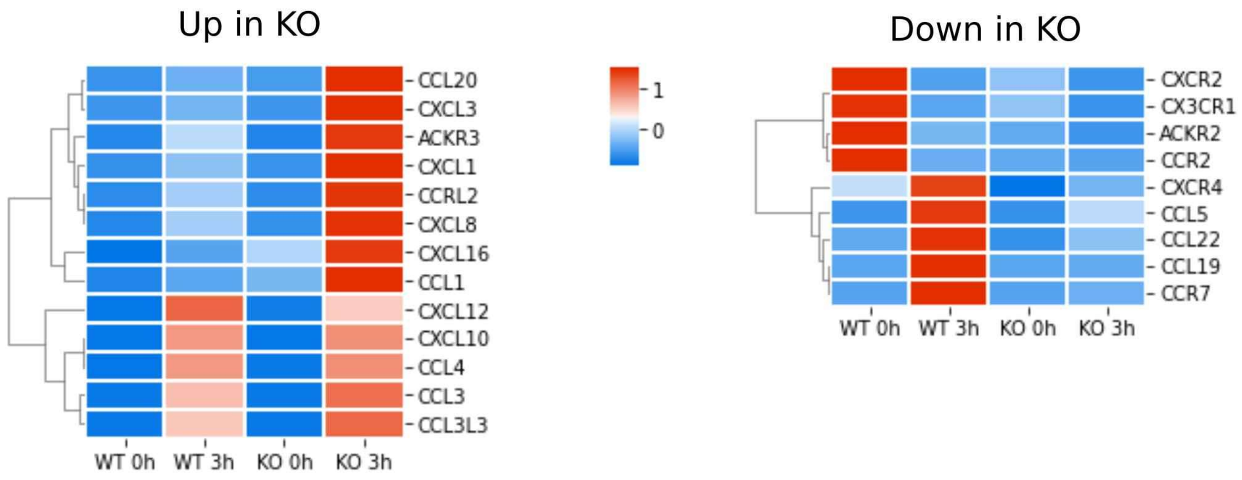

Supplementary Figure 6: Heatmaps showing genes DE (qvalule < 0.05) involved in Reactome "chemokine receptors bind chemokines" pathway either upregulated or downregulated in NFKB1$^{-/-}$ THP1, Z-score normalized.

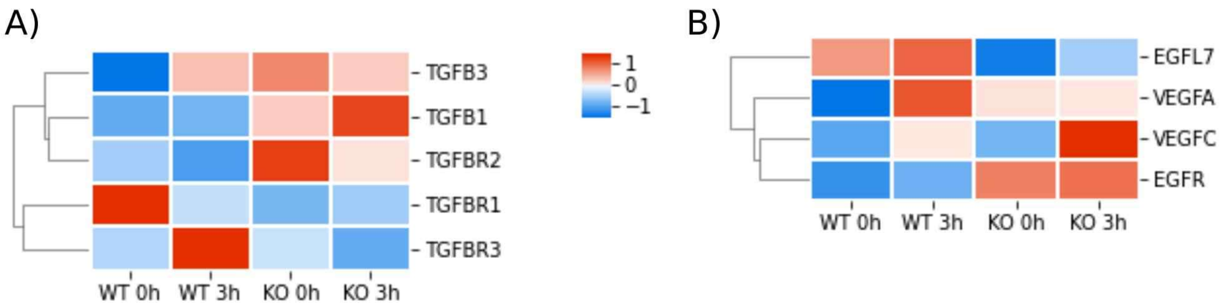

Supplementary Figure 7: EnrichR/Bioplanet query show an enrichment in TGF/VEGF/EGF pathways (qvalue <0.05). Heatmaps of **(A)** TGFB cytokines and receptors and **(B)** VEGF/EGF cytokines DE in RNAseq, Z-score normalized.

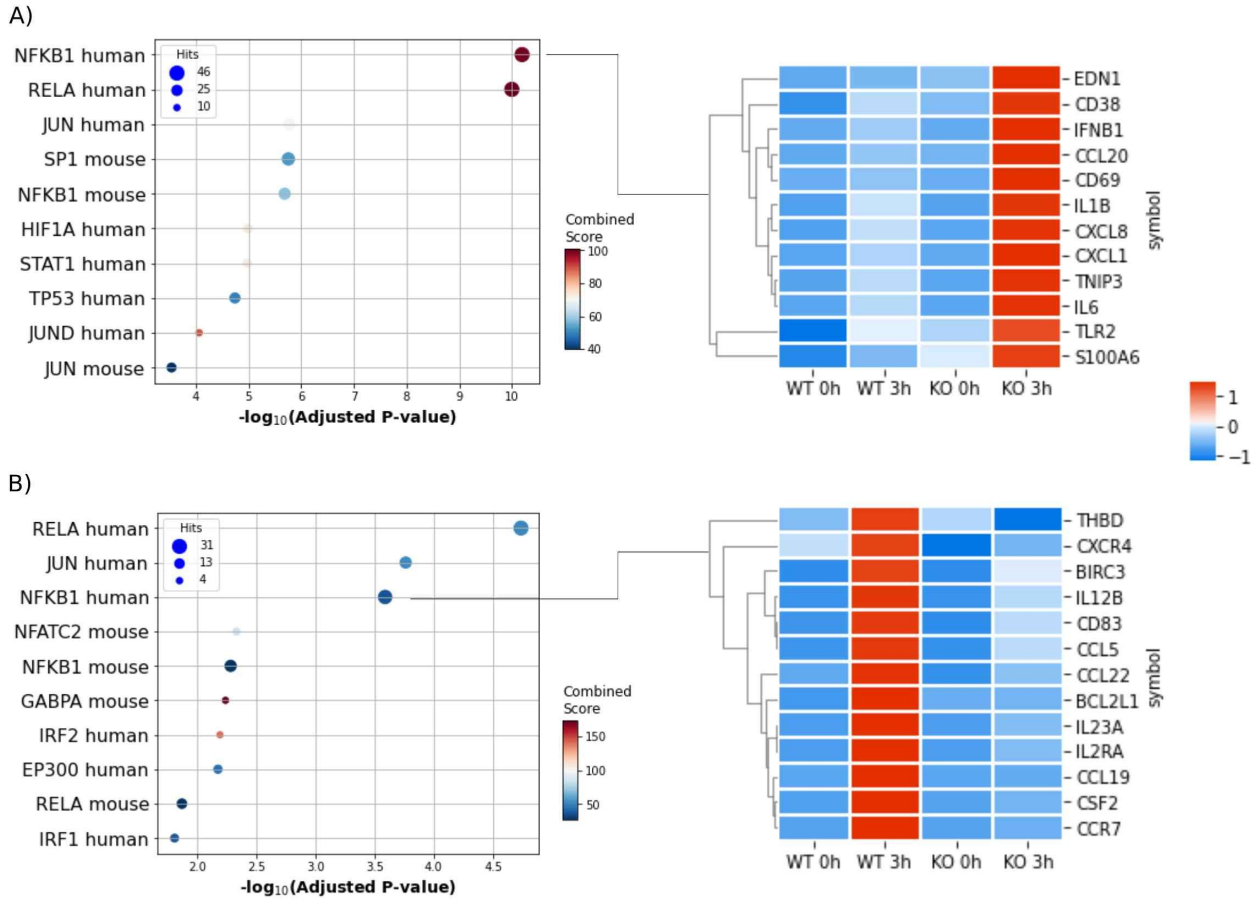

Supplementary Figure 8: Enrichr analysis querying TRRUST Transcription Factor database. **(A)** Transcription factors involved in the gene upregulation LPS-dependent controlled by p50 (on the left) and some of the genes controlled by p50 expression as heatmap (on the right). **(B)** Transcription factors involved in the gene downregulated LPS-dependent controlled by p50 (on the left) and some of the genes controlled by p50 expression as heatmap (on the right).
